# Experimental challenge of African green monkeys with contemporary Hendra virus isolates produces divergent clinical disease

**DOI:** 10.1080/22221751.2025.2544735

**Published:** 2025-08-06

**Authors:** Declan D. Pigeaud, Karla A. Fenton, Jacquelyn Turcinovic, Viktoriya Borisevich, Krystle N. Agans, Daniel J. Deer, Mack B. Harrison, Alyssa C. Fears, Natalie S. Dobias, Ahbishek N. Prasad, Ina L. Smith, David T. Williams, Courtney B. Woolsey, Christopher C. Broder, Robert W. Cross, Thomas W. Geisbert

**Affiliations:** a Galveston National Laboratory, University of Texas Medical Branch, Galveston, TX, USA; b Department of Microbiology and Immunology, University of Texas Medical Branch, Galveston, TX, USA; c Australian Centre for Disease Preparedness (ACDP), Commonwealth Scientific and Industrial Research Organization (CSIRO), Geelong, VIC, Australia; d Department of Microbiology and Immunology, Uniformed Services University, Bethesda, MD, USA

**Keywords:** Hendra virus, pathogenesis, animal models, paramyxoviruses, henipaviruses, neuroinfection, pathology, nonhuman primate

## Abstract

Hendra virus (HeV) is a medically important, zoonotic paramyxovirus that emerged over thirty years ago which causes severe, often fatal disease in humans and animals. There are presently no approved medical countermeasures to prevent or treat human HeV disease, although many are in various stages of development. Critical to the stringent evaluation of these experimental countermeasures are nonhuman primate models of HeV disease which accurately recapitulate the pathogenesis of human infection. The continued emergence of HeV since its initial discovery in 1994 has recently expanded to include a second genotype. Although this variant HeV produced fatal equine disease, its pathogenesis and lethality are unknown in humans. Here, we investigated the pathogenesis of clinically relevant and contemporary HeV isolates from genotype 1 (HeV/Australia/Horse/2008/Redlands) and genotype 2 (HeV-var/Australia/Horse/2015/Gympie) in the African green monkey (AGM) model of henipavirus disease. AGMs challenged with HeV genotype 1 (HeV-g1) or genotype 2 (HeV-g2) isolates via the combined intranasal/intratracheal route of exposure produced divergent survival outcomes, with four of five AGMs infected with the HeV-g2 isolate surviving. All five HeV-g1 infected subjects developed acute HeV disease which accurately recapitulated HeV pathogenesis reported in humans. Our findings revealed that HeV-g2 is less pathogenic than HeV-g1 in the AGM model and suggests that HeV-g2 may be less pathogenic in humans.

## Introduction

Hendra virus (HeV) is a highly pathogenic paramyxovirus (*Henipavirus hendraense*) that emerged in September 1994 in the suburb Hendra in Brisbane, Australia. The initial outbreak resulted in the deaths of 14 horses and their horse trainer, along with the nonfatal infection of seven additional horses and one other person [1]. Prior to this documented outbreak, the first known cases in humans and horses occurred several months prior, where a single person became ill after assisting in necropsies of two horses which were later shown to have died from HeV. This individual later experienced a relapsed fatal encephalitis 13 months later caused by HeV recrudescence [2]. HeV has since re-emerged in Australia 67 times with a total of 109 equine deaths (fatal or euthanized) along with four fatalities from seven human cases. Every known case of human HeV infection has involved horses; accordingly, individuals in the veterinary profession and those involved in the equine industry are at the highest risk for HeV exposure [3]. All documented human infections are positively correlated with exposure to the respiratory secretions, blood, or other bodily fluids of acutely infected horses [4]. Shortly after the initial spillover event, the *Pteropus* genus of flying foxes were identified as the natural reservoir of HeV, with replicating virus isolated from infected bats [5]. Several ecological factors including flying fox habitat loss and heat stress are predictive of HeV spillover events [6,7].

HeV is a negative-sense, single-stranded RNA virus possessing an approximately 18.2 kb genome encoding nine proteins: nucleoprotein (N), phosphoprotein (P), matrix (M), glycoproteins fusion (F) and attachment (G), polymerase (L), as well as (P) accessory proteins V, W, and C [8]. Glycoproteins (F) and (G) are essential for entry of susceptible host cells via (G) binding to surface receptors EphrinB2 or EphrinB3 [9]. Within the infected cell, accessory proteins V, W, and C modulate the host antiviral immune response by sequestering STAT proteins and preventing transcription of interferon-pathway associated genes.

Five equine HeV outbreaks have been responsible for the seven human cases to date, and all known human infections have been caused by isolates from genotype 1 (HeV-g1) which includes isolates HeV/Australia/Horse/1994/Hendra (HeV-prototype) and HeV/Australia/Horse/2008/Redlands (HeV-Redlands). The HeV-prototype strain was isolated from a fatal equine case in the 1994 outbreak and was the causative agent for the first four human infections of HeV which occurred in 1994, 1995, and 2005 resulting in three fatalities [1,2,10]. Subsequent HeV infections of humans are attributed to the HeV-Redlands strain, which caused three human infections from 2008 to 2009, two of which were fatal [11]. The initial outbreak from which the HeV-Redlands isolate was collected had high case fatality rates (CFR) in horses (80%) and humans (50%), and the clinical disease predominantly presented as neurological with minimal or no respiratory disease [11]. Horses experimentally infected with HeV-Redlands exhibited neurological signs of disease accompanied with respiratory signs including serous nasal discharge and dyspnoea which correlated to gross and histopathological lesions consistent with HeV infection.

HeV genotype 2 (HeV-g2) was first identified by RNA sequencing of *Pteropus poliocephalus* urine samples collected in 2013, and replicating virus was isolated from an archived plasma specimen of a fatal equine infection that occurred in 2015, yielding the HeV-var/Australia/Horse/2015/Gympie isolate [12,13]. The fatally infected horse exhibited clinical signs consistent with HeV disease; however, viral RNA was not detected with the HeV diagnostic qRT-PCR assay due to genomic variation. In 2021, an additional equine case of HeV-g2 was identified with a HeV-g2 specific qRT-PCR assay after the HeV-prototype diagnostic assay returned a negative result [14]. Several people reported close contact with the horse’s body following humane euthanasia; however, none reported illness consistent with HeV disease [14]. The HeV-g2 genome shares 89% and 83% nucleotide identity with the HeV-g1 prototype and Redlands isolates, respectively. Although there is some variation between amino acid sequence of the attachment glycoproteins of HeV-g1 and HeV-g2, HeV-g2 was potently neutralized by experimental therapeutic monoclonal antibody m102.4 and functional studies of the HeV-g2 glycoprotein revealed it is hypofusogenic compared to HeV-g1 [15,16].

The African green monkey (AGM) model of HeV disease is the gold standard for preclinical evaluation of experimental medical countermeasures; however, stringent assessment requires HeV isolates that are contemporary and which accurately recapitulate the clinical disease observed in humans. To date, no studies have been performed evaluating the pathogenesis of the HeV-g1 Redlands isolate or any HeV-g2 isolates in nonhuman primates (NHPs). Investigation of HeV pathogenesis in AGMs which employ endemic and emerging variants of HeV represent an understudied yet consequential area of study which precludes effective validation of vaccines or therapeutics necessary for reducing the public health burden of HeV. To address this gap in knowledge, we performed challenge studies in AGMs with HeV/Australia/Horse/2008/Redlands (HeV-g1) and HeV-g2 isolate HeV-var/Australia/Horse/2015/Gympie (HeV-g2) and report our findings on clinical disease, pathological lesions, and host response to infection.

## Methods summary

Detailed methods are delineated in the supplemental materials and methods. A brief, summarized version is listed here. The challenge stock of HeV-g1 was derived from the HeV/Australia/Horse/2008/Redlands isolate and was obtained from the European Virus Archive (EVAg, 023V-02990). The HeV-g2 challenge virus was propagated from the HeV-var/Australia/Horse/2015/Gympie isolate generously shared by collaborators from the Australian Centre for Disease Preparedness (ACDP). All challenge stocks were characterized by deep-sequencing, and two intermediate-frequency nonsynonymous substitutions were identified in the HeV-g2 stock (P) E687G (24%) and (L) L1485S (62%). *In vitro* replication kinetics were performed using Vero76 (ATCC CRL-1587), A549 (ATCC CCL-185) and extEqFL cells (ABM, T0095). Henipavirus titration and PRNT assays were performed on Vero76 cells as previously described [17]. Percent neutralization data was calculated in R v4.4.0., and the 50% neutralizing antibody titre (NT50) was calculated in GraphPad Prism v10.4.1 via 4-parameter logistic regression of percent neutralization values.

Two groups of five each healthy, adult AGMs were challenged with 5 × 10^5^ PFU of HeV-g2 or HeV-g1 divided evenly between the intranasal and intratracheal route and were monitored daily for disease progression. Clinical haematology and serum biochemistry assays were performed using Vetscan HM5 laser based haematologic analyzer (Zoetis) or Piccolo point-of-care analyzer (Abaxis) on whole blood or serum samples, respectively.

Necropsies were performed on all subjects and tissue samples were collected for histopathologic and IHC examination, immersion-fixed in 10% neutral buffered formalin, and processed for histopathology.

Whole blood samples containing HeV were inactivated in TRIzol LS (Invitrogen) or AVL buffer (Qiagen) and tissue samples were inactivated in RLT buffer (Qiagen). RNA was extracted following removal from BSL-4 containment according to manufacturer’s recommendation. Determination of viral RNA copies from blood or tissue specimens was performed by quantitative reverse transcriptase-polymerase chain reaction (qRT-PCR) using primers/probe targeting the (F) gene of HeV-g1 (Redlands) or HeV-g2, sequences are listed in supplemental materials and methods.

RNA extracted from TRIzol LS-inactivated whole blood samples was hybridized with the NHP_Immunology_V2 reporter and capture codesets containing ∼779 targets (Nanostring Technologies) for ∼24 h at 65˚C (∼149˚F) before being loaded into nCounter microfluidics cartridge which was immediately run on a Nanostring nCounter SPRINT Profiler.

IgG antibody titres from serum samples of HeV-g2 challenged AGMs were determined using NHP species ELISA kits from Alpha Diagnostic International for anti-HeV-G IgG (ADI RV-501120-1) and anti-NiV-G IgG (ADI NiV-015).

## Results

### In vitro characterization of viral growth kinetics

**Figure 1.**
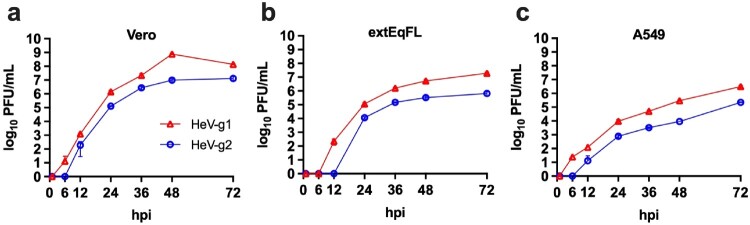
HeV-g1 and HeV-g2 growth kinetics in nonhuman primate, human, and equine cells. Individual points represent the means of assay duplicates, and error bars indicate ± SD. (a) Vero clone 76 cells. (b) A549 human lung epithelial cells. (c) Immortalized equine lung epithelial cells.

Replication kinetics between HeV-g1 and HeV-g2 were compared by infecting cell types derived from AGM, human, and equine tissue and serially titrating supernatants by plaque assay (Figure 1). The peak titre of HeV-g2 (7.1 log10 PFU/mL) on Vero cells was lower than HeV-g1 (8.9 log10 PFU/mL). This reduction in peak titre was also observed in human lung epithelial (A549) cells, with peak titres of HeV-g2 reaching 5.4 log10 PFU/mL compared to 6.5 log10 PFU/mL for HeV-g1. Lastly, to investigate the growth kinetics in the amplifying host of HeV, we infected immortalized equine lung epithelial (extEqFL) cells. The peak titre for HeV-g2 was 5.8 log10 PFU/mL and 7.3 log10 PFU/mL for HeV-g1. In all cell lines assessed, HeV-g2 grew more slowly than HeV-g1 and exhibited lower peak titres.

### Survival and clinical disease in African green monkeys experimentally challenged with HeV-g1 or HeV-g2 isolates

**Figure 2.**
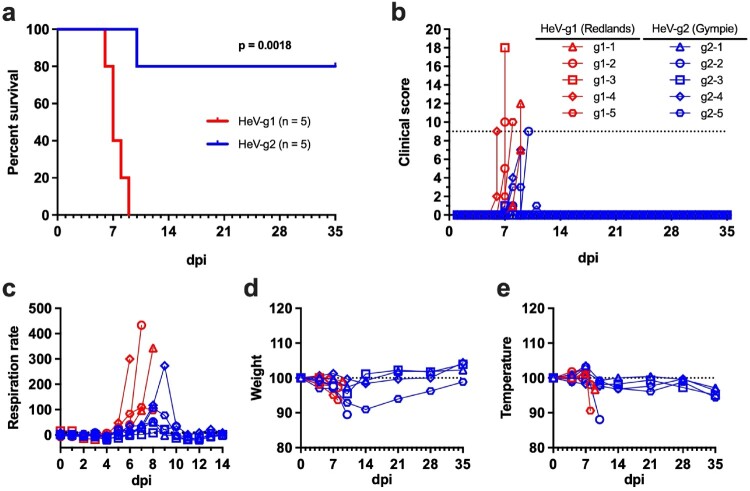
Survival and clinical disease of AGMs challenged with HeV-g1 or HeV-g2. (a) Kaplan-Meier survival curves for AGMs challenged with 5.0E5 PFU of HeV-g1 (red) and HeV-g2 (blue) via the it/in route. Statistically significant differences in curves were identified using a 2-tailed Mantel-Cox logrank test. (b) Clinical scores for HeV challenged AGMs; dotted line denotes the clinical scoring threshold requiring humane euthanasia. (c) Percent change of respiration rates from baseline as measured between day 0 and 14 DPI. Baseline respiration rates are calculated as the mean of respirations assessed from 0 to 4 DPI. (d) Weight change as percent of baseline. (e) Temperature change measured as percent of baseline. The weight and temperature recorded at 0 DPI was utilized as the baseline for each subject, dotted line represents 100% of baseline.

To compare the pathogenesis of these contemporary HeV isolates, we challenged two groups of n = 5 AGMs with either 5 × 10^5^ PFU of HeV-g1 (Redlands) or HeV-g2 (Gympie) via the combined intratracheal/intranasal (it/in) route. Infection with HeV-g1 produced a clinical illness characteristic of severe HeV disease and all subjects rapidly reached clinical scoring criteria mandating humane euthanasia by 9 days post infection (DPI) with a mean time to death of 7.4 DPI, ± 1 DPI (Figure 2(a,b)). Respiratory signs of disease observed in the HeV-g1 challenged cohort included dyspnoea (4/5) and abdominal breathing (3/5), which was accompanied by a rise in respiration rates beginning at 5 DPI (Figure 2(c)). Weight loss and decreases in temperature were observed in all AGMs challenged with HeV-g1 (Figure 2(d,e)). Two subjects exhibited neurological signs including ataxia, seizure, imbalance, and tremors (Table S1). Conversely, only a single subject in the HeV-g2 cohort met euthanasia criteria at 10 DPI, following the onset of dyspnoea and unresponsiveness which began at 9 DPI (Table S2, g2-2). The remaining four subjects survived until the scientific endpoint (35 DPI), and only exhibited nonspecific clinical signs including transient dyspnoea, mild inappetence, and low-grade fever which did not progress to severe respiratory distress or neurological disease. Haematological abnormalities starting at 4 DPI including thrombocytopenia and lymphocytopenia which progressively worsened until terminal endpoints in the HeV-g1 cohort; however, these parameters returned to baseline levels in HeV-g2-infected animals after 14 DPI (Table S1, S2). Serum levels of C-reactive protein (CRP) were elevated in all subjects following challenge; however, a temporal delay was observed in the HeV-g2 AGMs with peak serum CRP observed at 10 DPI compared to the 7 DPI or terminal timepoints in HeV-g1 cohort. Increases of serum creatinine were observed in all HeV-g1 challenged subjects and in subject g2-2. Levels of CRP and creatinine in the four AGMs that survived HeV-g2 infection returned to baseline by 21 DPI and remained there until the 35 DPI (Table S2).

### Viremia and viral load of HeV-g1 and HeV-g2 in experimentally infected AGMs

**Figure 3.**
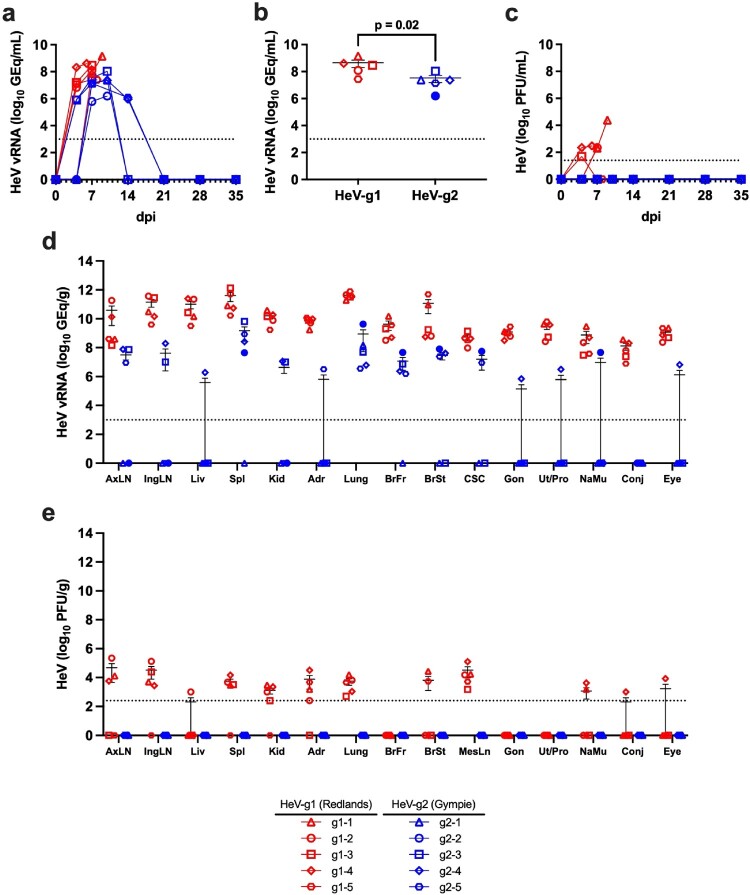
Viremia and HeV vRNA in challenged AGMs. (a) HeV vRNA in whole blood samples as determined by qRT-PCR. (b) Peak circulating vRNA quantities of HeV-g2 and HeV-g1 challenged AGMs, a 2-tailed Mann-Whitney U-test was performed to identify statistically significant differences between the distributions. (c) Plaque assay titration of HeV-g1 (red) or HeV-g2 (blue) in EDTA plasma samples from infected animals. (d) HeV vRNA detected in selected tissues of AGMs collected at necropsy by qRT-PCR. (e) Plaque assay titration of homogenates from tissues sampled at necropsy. For all assays, individual points represent the mean of two technical replicates and error bars indicate ± SEM. For (a), the 10 DPI sample was excluded for subject g2-5 due to sample extraction error. Dotted lines indicate the limit of detection (LOD) for the assay; 1000 GEq/mL or GEq/g for qRT-PCR, and 25 PFU/mL or 250 PFU/g for plaque assays. Sample where vRNA or plaques were not detected are plotted below the limit of detection fo each assay at a value of 1 to appear on the log axis. The solid blue circle indicates the 10 DPI terminal HeV-g2 subject. A lack of data plotted indicates that the tissue was not assayed for the given subject. AxLN, axillary lymph node; IngLN, inguinal lymph node; Liv, liver; Spl, spleen; Kid, kidney; Adr, adrenal gland; BrFr, brain frontal lobe; BrSt, brain stem; CSC, cervical spinal cord; Gon, gonad; UtPr, uterus/prostate; NaMu, nasal mucosa; Conj, conjunctiva; MesLN, mesenteric lymph node.

Viral RNA (vRNA) was extracted from whole blood samples and all subjects infected with HeV-g1 or HeV-g2 demonstrated capacity to support viral replication with HeV vRNA first detected in blood specimens at 4 DPI (Figure 3(a)). The peak circulating vRNA ranged from 7.4 to 9.2 log10 genome equivalents per millilitre (GEq/mL) for HeV-g1 and 6.2–8.0 log10 GEq/mL for HeV-g2, and this difference was statistically significant (Figure 3(b)). Peak vRNA load was typically observed at terminal timepoints for HeV-g1-infected subjects. Subject g2-2 (euthanized 10 DPI) had the lowest peak circulating vRNA of the HeV-g2 challenged cohort.

Replicating virus was detected in 3/5 blood plasma samples collected from HeV-g1-challenged AGMs starting at 4 DPI (Figure 3(c)). Titres ranged from 2.3 to 4.4 log10 plaque-forming units per millilitre (PFU/mL). No infectious virus was detected by plaque assay from plasma specimens in the HeV-g2-infected cohort at any point in the study (limit of detection 25 PFU/mL).

### Detection of HeV vRNA and replicating virus in tissues of infected AGMs

qRT-PCR detection of HeV-g1 and HeV-g2 vRNA from tissues collected at necropsy is reported as genome equivalents per gram of tissue (GEq/g). HeV-g1 vRNA was detected in the lungs (10.4–12.6 log10 GEq/g), lymphoid (7.5–12.3 log10 GEq/g), central nervous (8.5–11.7 log10 GEq/g), and urogenital (7.2–10.6 log10 GEq/g) tissues of all five AGMs (Figure 3(d)). Quantities of HeV-g1 vRNA were greatest in respiratory and lymphoid tissues, followed by the CNS and urogenital tract. HeV-g2 vRNA was detected in tissues assayed but at lower quantities and was not detected in all samples for all HeV-g2 infected AGMs. Copies of HeV-g2 vRNA were highest in the lungs (5.7–10.2 log10 GEq/g), followed by lymphoid (7.0–9.8 log10 GEq/g), urogenital (5.8–7.1 log10 GEq/g), and CNS (6.2–8.0 log10 GEq/g) tissues. Subject g2-2 (euthanized 10 DPI) had higher levels of HeV-g2 vRNA in the CNS of all other subjects in the cohort; however, the magnitude of these differences was less than 1.0 log10 GEq/g when compared survivor subjects with detectable HeV-g2 vRNA. Subject g2-2 generally had higher vRNA copies in other tissues as compared to survivors with the exception being lymphoid and urogenital tract tissues.

Replicating HeV in tissue homogenates was determined by plaque assay and is reported as plaque forming units per gram of tissue (PFU/g). HeV-g1 was detected in the respiratory and lymphoid tissues (Figure 3(e)). Respiratory viral titres ranged from 2.7 to 4.1 log10 PFU/g, and slightly higher peak titres were identified in the lymphoid tissues sampled (5.6 log10 PFU/g). HeV-g1 was not detected by plaque assay in the frontal lobe of the brain, gonads, or uterus/prostate, although titres were observed in the brain stem of two subjects (Figure 3(e)). HeV-g1 was detected in kidney tissue of 3/5 subjects, with titres ranging from 3.0 to 3.6 log10 PFU/g (Figure 3(e)). Replicating HeV-g2 was not detected in the tissues of any subjects within the HeV-g2 challenge cohort.

### Gross pathology findings in AGMs challenged with HeV-g1 or HeV-g2

**Figure 4.**
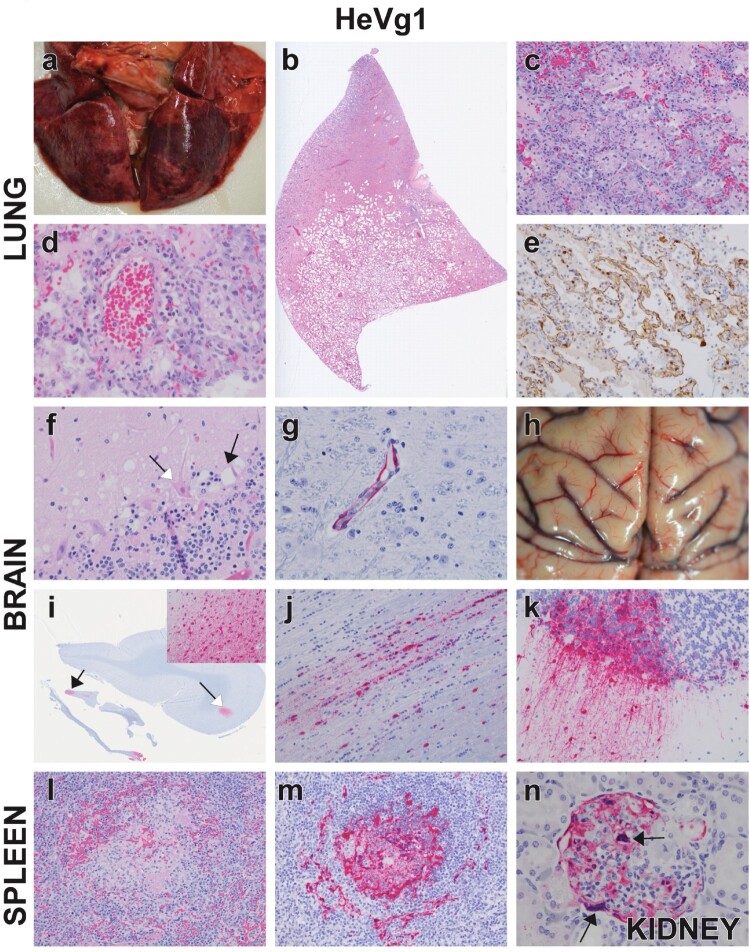
Representative gross and histopathologic lesions for HeV-g1 infected AGMs, for lung (a–e), brain (f–k), spleen (l,m) and kidney (n). (a) gross pathology, lungs, subject g1-4. Failure to collapse with patchy red and pink regions across all lung lobes (hemorrhagic interstitial pneumonia). (b) H&E, whole slide scan, lung, subject g1-4. Normal pulmonary architecture is obscured and/or destroyed by thickening of alveolar walls diffusely and locally extensive regions of alveolar flooding. (c) H&E, Lung, 20x, g1-4. Higher magnification of panel b, interstitial pneumonia with alveolitis characterized by expansion of alveolar septal walls with increased numbers of mononuclear inflammatory cells, congestion, and acellular pale eosinophilic material consistent with fibrin. (d) H&E, lung, 40x magnification, subject g1-4, vasculitis. (e) IHC, lung, 20x, subject g1-4. Extensive positive IHC labelling for HeV antigen (brown) of the alveolar septal walls and alveolar macrophages. (f) H&E, cerebellum, 40x, subject g1-1. Multifocal neuropil vacuolation (black arrow), with Purkinje cell degeneration/necrosis (white arrow). (g) IHC, brainstem, 40x, subject g1-3. Positive IHC labelling for HeV antigen (red) of endothelium within the neuroparenchyma. (h) Gross pathology, brain, subject g1-5. Meningeal congestion. (i) IHC, whole slide scan, brain (frontal lobe with olfactory tract), subject g1-1. Positive IHC labelling for HeV antigen (red) within the olfactory tract (black arrow) and focally within the frontal lobe (white arrow). (i inset) IHC, brain (frontal lobe), 20x, subject g1-1. Higher magnification of (i) (white arrow). Positive IHC labelling of HeV antigen (red) in a focal cluster of neuronal cells within the frontal lobe of the brain. (j) IHC, olfactory tract, 20x, subject g1-1. Higher magnification of (i) (black arrow). Positive IHC labelling for HeV antigen (red) is associated with increased numbers of satellite cells and neuronal cells located within the olfactory tract. (k) IHC, cerebellum, 20x, subject g1-2. Positive IHC labelling for HeV antigen (red) of cells within the molecular and granular layers in the cerebellum. (l) H&E, 20x, subject g1-2. Destruction of normal white pulp architecture with loss of lymphocytes, haemorrhage, and fibrin deposition. (m) IHC, spleen, 20x, subject g1-2. Positive IHC labelling for HeV antigen (red) of the splenic white pulp. (n) IHC, kidney, 40x, g1-1. Positive IHC labelling for HeV antigen (red) of the endothelium and syncytial cells (black arrows) of the renal glomerular tufts.

**Figure 5.**
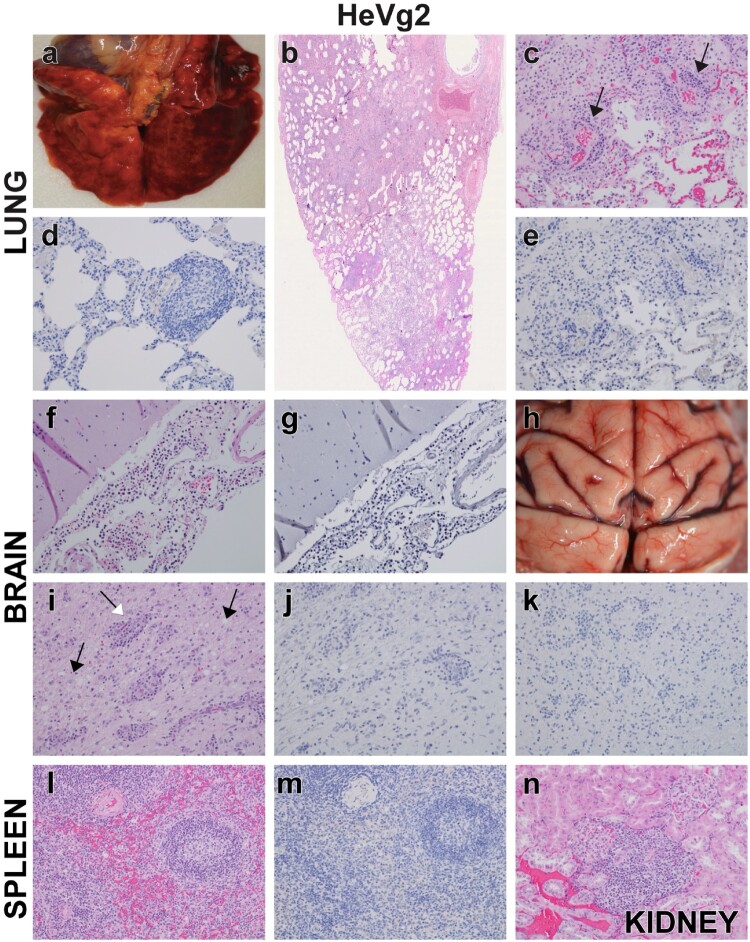
Representative gross and histopathologic lesions for HeV-g2 infected AGMs for lung (a–e), brain (f–k), spleen (l, m) and kidney (n). (a) Gross pathology showing lungs from g2-2 (euthanized 10 DPI) with lesions consistent with interstitial pneumonia. (b) H&E, lung, slide-scan, g2-2, normal pulmonary architecture is obscured by vascular infiltrates and thickened alveolar walls. (c) Higher magnification of panel b, with perivasculitis (arrows) and interstitial pneumonia. (d) IHC, lung, 20x, g2-4, perivasculitis and IHC negative (brown). (e) IHC, lung, 20x, g2-2 IHC negative (brown) of serial section of lung from panel c. (f) H&E, brain (meninges) 20x, g2-2, leptomeningitis. (g) IHC, brain (meninges), 20x, g2-2, IHC negative (brown). (h) Gross pathology, brain, subject g2-2. Meningeal congestion. (i) H&E, brainstem, 20x, subject g2-3. Multifocal perivascular cuffs (white arrow) associated with mild degeneration of the neuropil (black arrows) of the brainstem. (j) IHC, brainstem, 20x, g2-3, IHC negative (brown). (k) IHC, parietal lobe of the brain, 20x, g2-3, IHC negative (brown). (l) H&E, spleen, 20x, subject g2-2. Paucicellular splenic germinal centres, normal architecture. (m) IHC, spleen, 20x, subject g2-2 IHC negative (brown). (n) H&E, kidney, 20x, g2-3. Lymphohistiocytic interstitial infiltrates of the renal cortex.

All AGMs in the HeV-g1 cohort rapidly succumbed to disease and were humanely euthanized between 6 and 9 DPI. The most severe and common lesions involved the respiratory, central nervous, and lymphoid systems with all subjects exhibiting moderate to marked interstitial pneumonia (Figure 4(a)), meningeal congestion (Figure 4(h)), and lymphadenomegaly. The AGM challenged with HeV-g2 euthanized at 10 DPI (g2-2) displayed mild-to-moderate interstitial pneumonia, congestion of the meninges, and lymphadenomegaly (Figure 5(a,h)). Mild congestion of the lungs and meninges were noted in 2/4 surviving AGMs challenged with HeV-g2.

Histopathology and immunohistochemistry findings in tissues of HeV-g1 or HeV-g2 challenged AGMs.

Severe respiratory lesions located diffusely across all lung lobes were noted in all AGMs challenged with HeV-g1 (Figure 4(b)). Primary histopathological findings in the lung parenchyma included interstitial pneumonia with alveolitis characterized by expanded alveolar septae, increased numbers of mononuclear inflammatory cells, congestion, and fibrinous material (Figure 4(c)). Lesions of the large airway were generally less severe and consisted of minimal epithelial damage with accumulation of intraluminal, oedematous debris. Vasculitis of medium-to-small calibre vessels were noted throughout the lungs (Figure 4(d)). Collectively, histopathological lesions in the lungs aligned with the interstitial pneumonia and pleuritis noted at gross examination. Immunohistochemistry (IHC) for anti-henipavirus nucleoprotein (N) antigen colocalized with the lesions noted on H&E, with the alveolar septal walls and vascular endothelium being the most frequently positive structures (Figure 4(e)). Less frequently observed were IHC positive cells in the subepithelial stroma of larger airways, and positive respiratory epithelium within the pulmonary parenchyma or trachea was extremely rare.

In the HeV-g2-challenged cohort, pulmonary lesions were diffusely present and consisted of perivasculitis, vasculitis, and interstitial pneumonia (Figure 5(b,c,d)). Diffuse congestion, oedema, and cellular degeneration were all attributed to euthanasia and autolysis. The severity of pulmonary lesions was most pronounced in the subject euthanized at 10 DPI (g2-2), however no IHC positivity for HeV antigen was detected in the respiratory tract sections that were examined (Figure 5(d,e)).

Sections of neuronal tissues were evaluated for each AGM, with HeV antigen was detected in all CNS tissue regions in the HeV-g1 cohort. AGMs that succumbed to disease at 8 or 9 DPI (g1-5, g1-1) displayed the most extensive lesions; however, no overt lesions were noted on H&E for the two AGMs that succumbed early in the disease course (g1-3, g1-4) although IHC-positive endothelium was noted throughout the parenchyma of the CNS tissue examined (Figure 4(g)). AGM g1-2 (euthanized 7 DPI) had viral antigen detected in the endothelium of the brainstem, cerebellum, trigeminal ganglion, and dura mater (Figure 4(k)). IHC positivity was associated with increased numbers of satellite cells and neuronal cells located within the olfactory tract (AGM g1-1, Figure 4(j)), focal clusters of neurons with neuropil vacuolation in the frontal, parietal and temporal lobes, multifocal clusters of neurons within the cerebellum associated with vacuolar degeneration at the junction of the granular cell layer and molecular cell layer, and neurons within the pons of the brainstem (Figure 4(f,i,k)).

All sections of neuronal tissues evaluated for the HeV-g2-challenged AGMs were negative for HeV antigen by IHC (Figure 5(g,j,k)). Leptomeningitis was observed in subject g2-2 (euthanized 10 DPI, Figure 5(f,g)) and multifocal clusters of perivascular cuffs associated with mild degeneration of the neuropil were identified in surviving subjects g2-3, g2-4, and g2-5. No appreciable lesions were noted in the CNS of subject g2-1.

Viral antigen was detected by IHC in all examined lymphoid organs in the HeV-g1-challenged cohort. The spleen was the most consistently affected with loss of germinal centres and syncytial cell formation corresponding with IHC-positive cells within the germinal centre, marginal zone, and red pulp of the splenic follicles (Figure 4(m)). Expansion of lymphoid architecture was noted in peripheral lymph nodes and tonsil and was accompanied by syncytial cells within the germinal centre and histiocytic infiltrates. Germinal centre apoptosis and necrosis were observed in at least one of the lymphoid tissues in each NHP. Mononuclear cells were IHC positive within the germinal centre (including syncytial cells) and subcapsular sinuses of the lymph nodes and tonsil. Rarely the overlying epithelium of the tonsil was IHC positive.

HeV antigen was not detected in the lymphoid tissues for the HeV-g2-infected AGMs (Figure 5(m)). The splenic germinal centre for subject g2-2 was paucicellular but maintained normal architecture (Figure 5(l)), and no appreciable splenic lesions were noted for surviving AGMs. Of the remaining examined lymphoid tissues, normal architecture was observed with occasional lymphoid histiocytosis of the subcapsular sinuses.

Vascular lesions within the kidney were the most striking among the urogenital tissues of HeV-g1-infected AGMs and were characterized by multifocal obstructed glomeruli with fibrin thrombi and endothelial syncytial cells. Glomerular tuft endothelium and vessels throughout the kidney were IHC positive (Figure 4(n)). Minimal histiocytic inflammation in the submucosa of the urinary bladder colocalized with antigen positive mononuclear cells and endothelium for subject g1-2. IHC positivity was also noted in smooth muscle and endothelium of small calibre vessels in the rete testes, ovary, prostate, and uterus of several subjects. Lymphohistiocytic interstitial infiltrates of the renal cortex were observed in HeV-g2 subjects at 35 DPI (Figure 5(n)) but no other lesions or IHC positivity were noted. Additional pathology findings can be found in the Supplementary Data.

### Antibody response in survivors of heV-g2 challenge

Plasma and serum specimens from AGMs infected with HeV-g2 were assayed for by ELISA and plaque reduction neutralization tests (PRNTs). Subjects developed serum IgG antibodies to HeV (G), which also cross-reacted to related Nipah virus (NiV) (G) which was detected by ELISA. Peak ELISA titres reached 1:102,400 to HeV (G) and 1:51,200 to NiV (G) by 28 DPI (Figure S1a). Detection of neutralizing antibodies to HeV-g2 (Gympie), HeV-g1 (Redlands), and NiV strain Bangladesh by PRNT was performed with plasma specimens collected at 35 DPI and the 50% neutralizing antibody titre (NT50) was calculated (Figure S1b), with serum from all subjects assayed neutralizing at various levels.

Soluble markers of vascular inflammation and coagulation pathways detected in plasma of AGMs challenged with HeV-g1 or HeV-g2.

Plasma specimens collected from HeV-g1- or HeV-g2-infected AGMs were assayed by multiplex immunoassay to identify circulating proteins associated with vascular inflammation, thrombosis, and chemotaxis, after which the log2 fold change (log2FC) from baseline (0 DPI) was calculated (Figure S2). Following challenge, all AGMs exhibited marked log2FC increases in proteins involved in vascular damage, coagulation pathway activation, and immune cell recruitment at multiple timepoints. Statistically significant differences of extrinsic and intrinsic coagulation pathway proteins were identified between viral challenge groups were noted at both 7 and 10 DPI. Significant differences in log2FC values for P-selectin, PSGL-1, and sCD40L were observed between the HeV-g2 and HeV-g1 AGMs at acute disease timepoints. In general, HeV-g1-infected AGMs exhibited greater peak log2FC values for analytes associated with chemotaxis (Eotaxin, IL-8, IP-10, MCP-1, and MIP-1beta) whereas HeV-g2 infection produced increased levels of fibrinolysis and endothelial damage markers (tissue factor, factor IX, P-selectin, tPA, sCD40L, PSGL-1, PAI-1). Peak log2FC values of vascular damage markers were most commonly observed at 14 and 21 DPI for HeV-g2 challenged AGMs.

Targeted transcriptomics of whole blood samples in AGMs challenged with HeV-g1 or HeV-g2.

**Figure 6.**
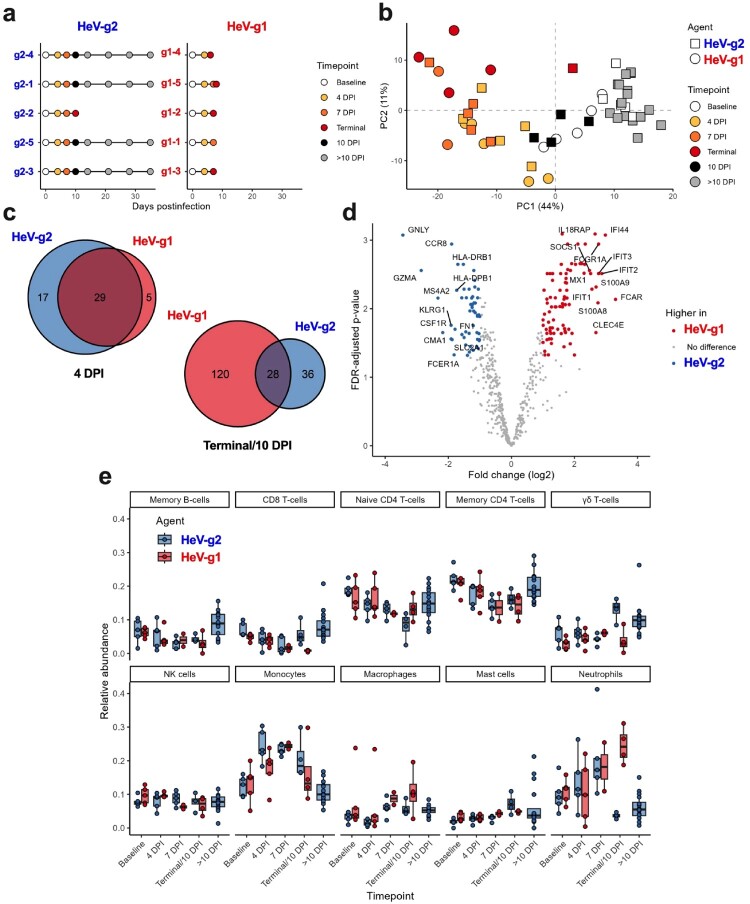
Transcriptional responses of AGMs challenged with HeV-g1 or HeV-g2. (a) Sampling plot of whole-blood RNA samples collected and analysed by Nanostring. (b) PCA dimension reduction of assessed samples by agent (HeV-g2, squares; HeV-g1, circles) and timepoint (baseline, 0, 4, 6, terminal, 10 DPI, > 10 DPI). (c) Venn diagram comparing significant DEGs at 4 DPI and 10/terminal DPI timepoints between the HeV-g2 (blue) and HeV-g1 (red) cohorts. (d) Volcano plot depicting log2 fold change for the top DEGs between HeV-g1 and HeV-g2 challenged cohorts at acute disease timepoints (10 DPI for HeV-g2, terminal for HeV-g1). (e) Differential cell quantitation via CIBERSORT [18] to estimate relative abundances of immune cells in the blood of HeV-g2 and HeV-g1 infected AGMs.

The host immune responses to HeV-g1 or HeV-g2 infection were investigated by targeted transcriptomics on whole blood samples (Figure 6(a)). Dimension reduction via principal component analysis (PCA) revealed variation was influenced primarily by timepoint and not by challenge virus (Figure 6(b)). There was substantial overlap in differentially expressed (DE) genes at 4 DPI relative to baseline (Figure 6(c)), and direct comparison between cohorts detected no statistically significant DE genes. The host response to infection subsequently diverged when 10 DPI in HeV-g2 challenged animals was compared to terminal timepoints in HeV-g1 challenged animals (Figure 6(d)). Upregulated transcripts at 10 DPI in HeV-g2 challenged AGMs included those related to antigen presentation (*HLA-DRB1, HLA-DPB1*), T/NK cell activation (*IL15, GNLy, CCR8*), and monocyte migration and inflammation (*LGALS3, CCR2*). Upregulated transcripts in HeV-g1-challenged AGMs at terminal timepoints were generally associated with pathways implicated in innate antiviral immunity including ISGs (*IFI44, IFI35, IFIT1, IFIT2*), PRRs (*DDX58, OAS3, IFIH1*), and protein kinase R (*EIF2AK2*). Additional upregulated transcripts were indicative of the aberrant immune response to HeV-g1 infection and are associated with both proinflammatory (*IL1B, IL18RAP, NCR1, IL18R1*) and anti-inflammatory (*IL1RN, TNFAIP3, MMP9, SOCS3*) signalling.

To investigate potential differences in the circulating immune cell populations from whole blood samples between the HeV-g1 and HeV-g2 challenged cohorts, we utilized the CIBERSORT [18] analytical tool on the targeted transcriptomics data (Figure 6(e)). No statistically significant differences were identified between the abundance of inferred immune cell populations; however, the relative abundance of neutrophils at terminal timepoints for HeV-g1 infected AGMs trended higher than subjects in the HeV-g2 cohort. Several inferred T-cell populations including CD8 T-cells and gamma-delta T-cells were more abundant in the HeV-g2 cohort at the 10 DPI/terminal timepoint.

## Discussion

Over thirty years have elapsed since the emergence of HeV in 1994. To date, there are no approved vaccines to protect human populations at risk for HeV infection, nor are there medical countermeasures capable of successfully treating the fatal recrudescent encephalitis that can occur following resolution of acute disease. The sporadic nature of human HeV cases and the biosafety considerations required for handling specimens has limited the depth of investigation of HeV pathogenesis. The AGM model of henipavirus disease accurately recapitulates the probable natural routes of exposure, clinical disease, and pathological lesions of HeV disease in humans. Studies of HeV in the AGM species is required for the accurate assessment of medical countermeasures and to provide a reference for the future use of alternative regulatory approval pathways, such as FDA's Animal Rule. The objective of the experimental challenge studies described herein was to formally compare the pathogenesis of contemporary HeV isolates from genotype 1 and genotype 2 in AGMs, which will inform future countermeasure development efforts.

Experimental infection of the AGMs with HeV-g1 and HeV-g2 resulted in drastically different survival outcomes. Contrary to the severe, uniformly fatal disease observed in HeV-g1 infection, HeV-g2-infection of AGMs produced a clinical disease characterized by moderate respiratory signs, and four of five subjects survived until the scientific endpoint of the study. Given the severe disease observed in the two documented equine cases and the genetic similarity of HeV-g2 to HeV-g1, we did not anticipate that HeV-g2 would be less pathogenic in the AGM model [12–14]. The high degree of similarity in the serum biochemistry, haematology, vascular inflammation markers, and whole blood transcriptomics suggests that the early response to HeV-g2 and HeV-g1 challenge is largely the same; however, the comparatively lower peak levels of viral genomes detected in the blood of the HeV-g2 infected AGMs indicated that reduced viral replication kinetics could contribute to the observed differences in survival outcomes. This is further substantiated by the lack of detectable replicating HeV-g2 from plasma or tissues of experimentally infected AGMs. Additionally, the pathological lesions from the HeV-g2 subject euthanized at 10 DPI did not include positive immunostaining for HeV antigen nor the clinically relevant lesion of syncytial cells in any of the sections examined, suggesting that replicating HeV-g2 was cleared relatively quickly. Henipavirus antigen detected by IHC was documented in the vessel walls of cardiac tissue sampled from a flying fox retrospectively identified to be infected with HeV-g2; however, antigen positivity was not reported for the equine HeV-g2 infections due to lack of sampling [12]. Although no signs of neurological disease were observed in the HeV-g2-challenged AGMs, 4/5 subjects had inflammatory lesions in the brain associated with small calibre vessels. Our analysis of circulating markers for vascular inflammation revealed large fold change increases of mediators for endothelial damage and thrombosis following inoculation with HeV-g2, coagulopathies which align with the vasculitis we observed in tissues. The extended duration of these coagulopathies out to 14 and 21 DPI is likely an artefact of the animals surviving acute HeV-g2 challenge, as those infected with HeV-g1 succumbed to disease prior to these timepoints. sCD40L, released by activated platelets and T-cells has been implicated in blood–brain barrier permeability breakdown and enhanced macrophage adhesion to endothelial cells, all of which may have contributed to the inflamed state of CNS tissues we observed in the experimentally infected AGMs [19]. Given the concern of recrudescent encephalitis in survivors of HeV disease [20], further investigation should be performed to examine the role of sCD40L and other mediators of vascular damage during HeV infection.

We performed several *in vitro* studies to compare the growth kinetics of HeV-g1 and HeV-g2 (Figure 1), which revealed that HeV-g2 exhibits slower growth in equine, human, and NHP cells. Recently work has revealed that HeV-g2 glycoproteins produce fewer syncytia and that the F glycoprotein specifically is hypofusogenic when compared to HeV-g1 [16]. However, the correlation between pathogenesis and syncytial cell formation in HeV disease has not been substantiated in the field. Given the relevance of this lesion to the identification and diagnosis of HeV infection, this should be investigated further in both *in vitro* and *in vivo* studies. Furthermore, future in vitro work is necessary in target cell types known to express HeV Ephrin receptors including endothelial cells from pulmonary and neuronal tissues. This may help to better understand the contribution of infection in these cell types without the interruption circulating host immune responses to infection.

In summary, our results show a significant difference in the lethality of HeV isolates from genotype 1 and 2 in AGMs. Experimental infection of AGMs with HeV genotype 1 (isolate HeV/Australia/Horse/2008/Redlands) produced uniformly lethal disease characterized by the clinical and pathological hallmarks observed in humans. Conversely, infection of with HeV genotype 2 (isolate HeV-var/Australia/Horse/2015/Gympie) produced mild to subclinical disease, with only one succumbing to infection. The findings of this study corroborate the described severe disease caused by the HeV-g1 Redlands isolate in humans and horses and indicate that HeV genotype 2 isolates may be less pathogenic in primate species.

## Supplementary Material

HeVg2_g1 manuscript EMI supplemental_29Jul2025_final clean.docx
