# Supplementary material for: Experimental challenge of African green monkeys with contemporary Hendra virus isolates produces divergent clinical disease
Source: Emerg Microbes Infect. 2025 Aug 6;14(1):2544735. doi: 10.1080/22221751.2025.2544735 (PMC12377146; doi:10.1080/22221751.2025.2544735)
Supplement: HeVg2_g1 manuscript EMI supplemental_29Jul2025_final clean.docx [file TEMI_A_2544735_SM4195.docx]

Supplementary Data

Table S1. Clinical pathology of AGMs after challenge with Hendra virus/Australia/Horse/2008/Redlands (HeV-g1)

| **Subject** | **Sex** | **Clinical Illness*** | **Clinical Pathology*** |
| --- | --- | --- | --- |
| g1-1 | Male | Increased respirations (d7,8); abdominal breathing (d9); recumbency (d9); Subject euthanized (d9). | Lymphocytopenia (d7,9); thrombocytopenia (d9); monocytopenia (d7); monocytosis (d9); neutropenia (d4); neutrophilia (d9); eosinopenia (d7); basopenia (d7,9); hyperglycemia (d9); hypoalbuminemia (d9); ↑CRE (d9); ↑AST (d9); ↑CRP (d9). |
| g1-2 | Male | Dyspnea (d7); abdominal breathing (d7); depression (d7); Subject euthanized (d7). | Thrombocytopenia (d7); lymphocytopenia (d4,7); monocytopenia (d7); neutrophilia (d7); basopenia (d7); hypoamylasemia (d7); ↑CRP (d7). |
| g1-3 | Male | Recumbency (d7); seizure (d7); Subject euthanized (d7). | Monocytosis (d4,7); neutropenia (d4); neutrophilia (d7); eosinopenia (d4,7); basophilia (d7); hyperglycemia (d7); ↑CRE (d7); ↑↑CRP (d7). |
| g1-4 | Male | Dyspnea (d6); abdominal breathing (d6); hunched posture (d6); Subject euthanized (d6). | Thrombocytopenia (d6); lymphocytopenia (d6); neutrophilia (d6); eosinopenia (d4); basopenia (d4,6); hyperglycemia (d6); hypoproteinemia (d6); hypoalbuminemia (d6); ↑AST (d6); ↑CRP (d6). |
| g1-5 | Female | Dyspnea (d6-8); hypothermia (d8); recumbency (d8); ataxia (d8); tremors (d8); imbalance(d8); Subject euthanized (d8). | Thrombocytopenia (d7,8); lymphocytopenia (d7,8); neutrophilia (d7,8); eosinopenia (d8); basopenia (d7,8); hyperglycemia (d8); hypoproteinemia (d8); hypoalbuminemia (d8); hypoamylasemia (d8); ↑CRP (d7); ↑↑CRP(d8). |
| *Days after Hendra virus/Australia/Horse/2008/Redlands (HeV-g1) challenge in parentheses. All reported findings are in comparison to baseline (day of challenge, i.e. d-0) values. Decreased appetite is defined as some food but not all food consumed from the previous day. Anorexia is defined as no food consumed from the previous day. Fever is defined as a temperature more than 2.5°F (~1.4°C) over baseline, or at least 1.5°F (~0.83°C)over baseline and ≥ 103.5°F (~39.7°C). Hypothermia is defined as a temperature ≤3.5°F (~1.9°C) below baseline. Lymphocytopenia, monocytopenia, erythrocytopenia, thrombocytopenia, neutropenia, eosinopenia, and basopenia are defined by a ≥35% drop in numbers of lymphocytes, monocytes, erythrocytes, platelets, neutrophils, eosinophils, or basophils, respectively. Lymphocytosis, monocytosis, neutrophilia, eosinophila, and basophilia are defined by a 100% or greater increase in numbers of lymphocytes, monocytes, neutrophils, eosinophils, and basophils, respectively. Hyperglycemia is defined as a 100% or greater increase in levels of glucose. Hypoglycemia is defined by a ≥25% decrease in levels of glucose. Anemia is defined as a concurrent ≥25% decrease in erythrocyte count, HCT, and HGB. Hypoalbuminemia is defined by a ≥25% decrease in levels of albumin. Hypoproteinemia is defined by a ≥25% decrease in levels of total protein. Hypoamylasemia is defined by a ≥25% decrease in levels of serum amylase. Hypocalcemia is defined by a ≥25% decrease in levels of serum calcium. Increases in ALT, AST, ALP, CRE, CRP, HCT, and HGB were graded on the following scale: ↑ = 1-5-fold, ↑↑ = >5-10 fold, ↑↑↑ = >10-20 fold, ↑↑↑↑ = >20-fold, ↓ = ≥50% decrease. Fold change calculated as fold change from baseline. (BUN) blood urea nitrogen, (ALT) alanine aminotransferase, (AST) aspartate aminotransferase, (ALP) alkaline phosphatase, (CRE) Creatinine, (CRP) C-reactive protein, (HCT) hematocrit, (HGB) hemoglobin. | | | |

Table S2. Clinical pathology of AGMs after challenge with HeV/Australia/Horse/2015/Gympie (HeV-g2)

| **Subject** | **Sex** | **Clinical Illness*** | **Clinical Pathology*** |
| --- | --- | --- | --- |
| g2-1 | Male | Fever (d7); Subject euthanized at study endpoint (d35). | Lymphocytopenia (d4,7); lymphocytosis (d14,21); monocytosis (d14,28); neutrophilia (d7,14); eosinopenia (d10); basopenia (d4,10); basophilia (d21); ↑ALT (d21). |
| g2-2 | Female | Fever (d7); dyspnea (d10); hypothermia (d10); Subject euthanized (d10). | Thrombocytopenia (d4); lymphocytopenia (d10); monocytosis (d7); neutrophilia (d7,10); eosinopenia (d4); basopenia (d4); ↓ALT (d7,10); ↑CRE (d7,10). |
| g2-3 | Female | Decreased appetite (d0,1,3-13, 15,17,22); anorexia (d8); Subject euthanized at study endpoint (d35). | Thrombocytopenia (d10); lymphocytosis (d14,21); monocytosis (d4,14,21,35); neutropenia (d35); eosinopenia (d4,7,10); basopenia (d7,10); ↑ALT (d35); ↑AST (d35); ↑CRP (d7,10,28). |
| g2-4 | Male | Decreased appetite (d9-11); hunched posture (d8-10); moderate dyspnea (d8,9); cough (d9); weakness (d13); Subject euthanized at study endpoint (d35). | Thrombocytopenia (d7,10); lymphocytosis (d14); monocytosis (d10,14); neutrophilia (d4,7,10,14,21,28); basopenia (d7,10); ↑ALT (d21,28); ↑CRP (d28). |
| g2-5 | Female | Decreased appetite (d7-16, 18); anorexia (d9); mild dyspnea (d8,9); hunched posture (d9); Subject euthanized at study endpoint (d35). | Thrombocytopenia (d7,10,28); lymphocytopenia (d4,28); monocytopenia (d28); neutropenia (d4,28,35); eosinopenia (d10,28,35); basopenia (d4,10,21,28,35); ↓ALT (d14); ↓ALP (d10); ↑CRP (d10). |
| *Days after Hendra virus/Australia/Horse/2015/Gympie (HeV-g2) challenge in parentheses. All reported findings are in comparison to baseline (day of challenge, i.e. d-0) values. Decreased appetite is defined as some food but not all food consumed from the previous day. Anorexia is defined as no food consumed from the previous day. Fever is defined as a temperature more than 2.5°F (~1.4°C) over baseline, or at least 1.5°F (~0.83°C)over baseline and ≥ 103.5°F (~39.7°C). Hypothermia is defined as a temperature ≤3.5°F (~1.9°C) below baseline. Hypothermia is defined as a temperature ≤3.5°F (~1.9°C) below baseline. Lymphocytopenia, monocytopenia, erythrocytopenia, thrombocytopenia, neutropenia, eosinopenia, and basopenia are defined by a ≥35% drop in numbers of lymphocytes, monocytes, erythrocytes, platelets, neutrophils, eosinophils, or basophils, respectively. Lymphocytosis, monocytosis, neutrophilia, eosinophila, and basophilia are defined by a 100% or greater increase in numbers of lymphocytes, monocytes, neutrophils, eosinophils, and basophils, respectively. Hyperglycemia is defined as a 100% or greater increase in levels of glucose. Hypoglycemia is defined by a ≥25% decrease in levels of glucose. Anemia is defined as a concurrent ≥25% decrease in erythrocyte count, HCT, and HGB. Hypoalbuminemia is defined by a ≥25% decrease in levels of albumin. Hypoproteinemia is defined by a ≥25% decrease in levels of total protein. Hypoamylasemia is defined by a ≥25% decrease in levels of serum amylase. Hypocalcemia is defined by a ≥25% decrease in levels of serum calcium. Increases in ALT, AST, ALP, CRE, CRP, HCT, and HGB were graded on the following scale: ↑ = 1-5-fold, ↑↑ = >5-10 fold, ↑↑↑ = >10-20 fold, ↑↑↑↑ = >20-fold, ↓ = ≥50% decrease. Fold change calculated as fold change from baseline. (BUN) blood urea nitrogen, (ALT) alanine aminotransferase, (AST) aspartate aminotransferase, (ALP) alkaline phosphatase, (CRE) Creatinine, (CRP) C-reactive protein, (HCT) hematocrit, (HGB) hemoglobin. | | | |

#
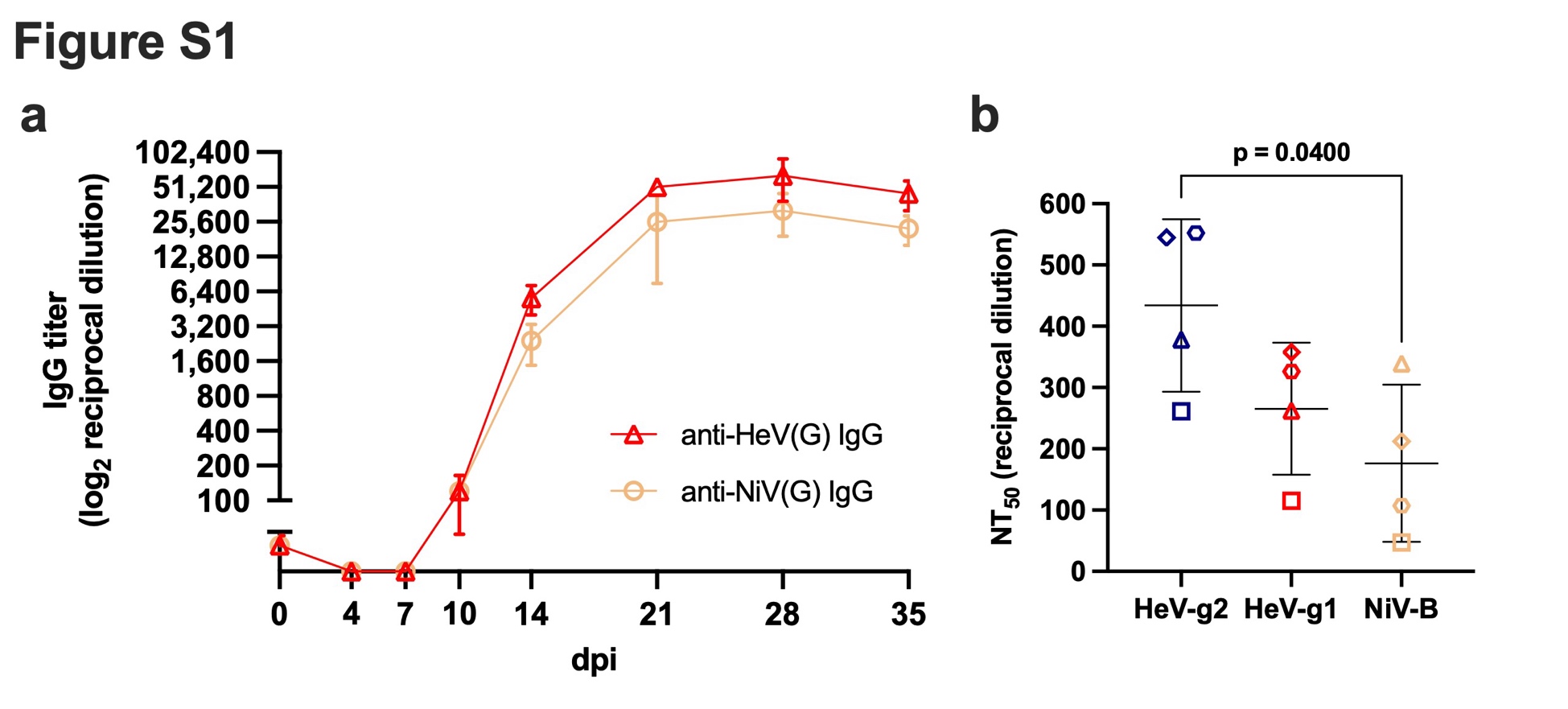


Figure S1: Antibody responses of AGMs challenged with HeV-g2. (a) IgG antibodies specific to the attachment glycoprotein (G) of HeV-g1 (prototype isolate) or Nipah virus strain Bangladesh (NiV-B) in serum sampled at indicated timepoints, determined by indirect ELISA (n = 4 AGMs per antigen per timepoint). (b) 50% neutralizing plasma antibody titres (NT50) of convalescent plasma sampled from HeV-g2 challenged subjects at 35 DPI against authentic HeV-g2, HeV-g1, and NiV-B viruses assayed by PRNT. A Friedman test with Dunn’s multiple comparison post-hoc test was applied to identify statistically significant differences between NT50 titres. A lack of significance plotted indicates comparison was not statistically significant. For all plots, individual points represent the mean of two sample replicates, horizontal bars indicate means and error bars represent ±SD.


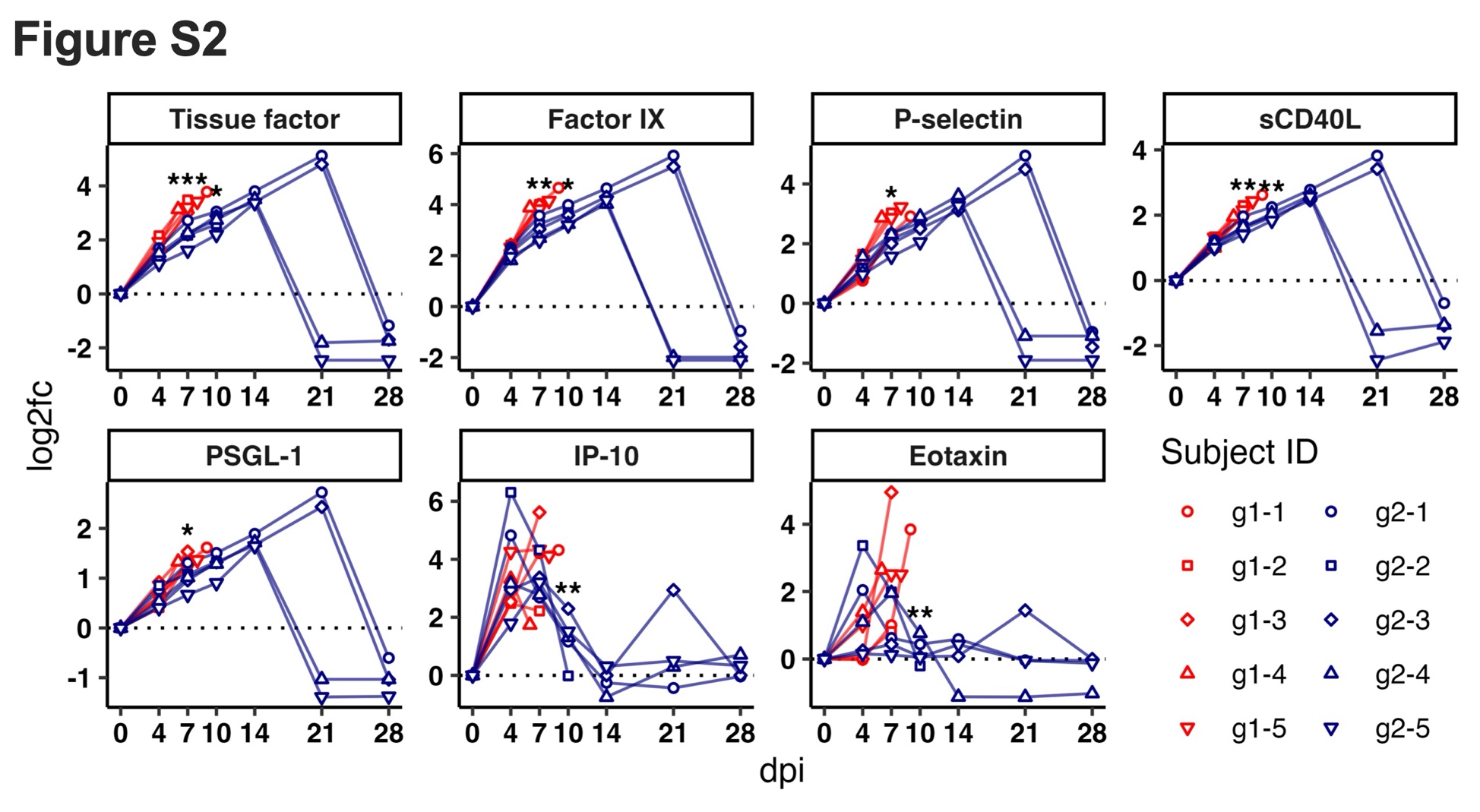


Figure S2: Selected protein markers implicated in vascular inflammation, chemotaxis, and coagulation pathways detected in plasma specimens of AGMs challenged with HeV-g1 or HeV-g2. Values are represented as log2 fold change (log2FC) of concentrations (pg/mL) from baseline (0 DPI) until 28 DPI. Individual points represent the mean of two technical replicates. Significant differences determined by repeated measures ANOVA with Tukey’s multiple comparison test; p<0.001 (***), p<0.01 (**), p<0.05 (*). The HeV-g1 terminal AGM sample collected at 6 DPI was grouped with day 7 for analysis, and terminal samples collected at days 8 and 9 were compared to HeV-g2 samples from 10 DPI. PSGL-1, P-selectin glycoprotein ligand-1; IP-10, interferon-gamma inducible protein 10 (CXCL10).

# Data availability

All study data are included in the main text or the supporting information. Nanostring transcriptomic data is available via NCBI GEO (submission in progress). Bioinformatic code is available on GitHub ([https://github.com/geisbert-lab/hev-g2-redlands](https://nam11.safelinks.protection.outlook.com/?url=https%3A%2F%2Fgithub.com%2Fgeisbert-lab%2Fhev-g2-redlands&data=05%7C02%7Cddpigeau%40UTMB.EDU%7C7d142188e6c341a7a94808dd7e011390%7C7bef256d85db4526a72d31aea2546852%7C0%7C0%7C638805265281197482%7CUnknown%7CTWFpbGZsb3d8eyJFbXB0eU1hcGkiOnRydWUsIlYiOiIwLjAuMDAwMCIsIlAiOiJXaW4zMiIsIkFOIjoiTWFpbCIsIldUIjoyfQ%3D%3D%7C0%7C%7C%7C&sdata=sNoshN59N4evaKK%2B4HlJy4I%2FZwUOPuiuXv%2BoM7QVsKA%3D&reserved=0)) and via Zenodo (DOI: 10.5281/zenodo.15238546). All other datasets used to produce this paper are available from the Corresponding Author upon request. Any additional information required to reanalyse the data reported in this paper is available from the Corresponding Author upon request.

# Supplemental Materials and Methods

## Study oversight

All study protocols described were approved by the UTMB Institutional Animal Care and Use Committee (IACUC) which were compliant with UTMB Institutional Biosafety Committee (IBC) guidelines under BSL-4 containment. UTMB animal facilities used in this work are accredited by the Association for Assessment and Accreditation of Laboratory Animal Care International and adhere to principles specified in the eighth edition of the Guide for the Care and Use of Laboratory Animals, National Research Council.

## Nonhuman primate challenge

A total of ten healthy adult African green monkeys (*Chlorocebus aethiops*) were randomly assigned into two groups of five animals each. Animals were anesthetized on study day 0 with an intramuscular injection of ketamine and inoculated with 5 x 10^5^ PFU of HeV-g2 or HeV-g1 divided evenly between the intranasal and intratracheal route. The duration of the NHP study was 35 days. All 10 AGMs were monitored daily and scored for disease progression with an internal henipavirus humane endpoint scoring sheet approved by the UTMB Institutional Animal Care and Use Committee (IACUC). The scoring changes measured from baseline included posture and activity level, attitude and behaviour, food intake, respiration, and disease manifestations, such as central nervous system disorders. A score of ≥ 9 indicated that an animal met the criteria for euthanasia.

## Deep sequencing of viral isolates

Reads aligning to the African green monkey genome were discarded (genome assembly ChlSab1; Bowtie2 v2.3.4.3[24]), and reads that did not align were metagenomically classified with Kraken2 v2.0.7-beta[25] to detect adventitious agents and other contaminants. Non-host reads were then aligned to the appropriate viral reference with Bowtie2 v2.3.4.3[24] (NCBI AY988601.1). PCR duplicates were masked before calculating coverage, assembling a consensus, and calling single nucleotide variants. Alignment processing, duplicate masking, and consensus building were accomplished via Samtools v1.20[26]. SNVs were called with LoFreq v2.1.3.1[27] and annotated in R v4.4.0 using the Biostrings[28] library.

## Viruses

The HeV Redlands stock is derived from the HeV/Australia/Horse/2008/Redlands isolate (NCBI accession HM044317) and was obtained from the European Virus Archive (EVAg, 023V-02990). The challenge virus stock was passaged five times on Vero76 cells. Supernatants were collected and stored at -80°C (-112°F) in ~1mL aliquots. No nonsynonymous mutations were detected at >5% relative abundance. The HeV genotype 2 stock used for challenge experiments was propagated from the HeV-var/Australia/Horse/2015/Gympie isolate (NCBI accession MZ318101), which was generously shared by collaborators from the Australian Centre for Disease Preparedness (ACDP). The stock virus was passaged once on Vero76 cells by our laboratory. Two intermediate-frequency nonsynonymous substitutions were identified in (P) E687G (24%) and (L) L1485S (62%). The NiV Bangladesh stock used for *in vitro* virus neutralization assays was derived from fatal human isolate 200401066. No detectable mycoplasma or endotoxin was present in either of the HeV challenge stocks.

## Cells

Vero76 cells were obtained from the American Type Culture Collection (ATCC, CRL1587) and maintained in EMEM (ATCC, 30-2003) containing 10% heat inactivated Foetal Bovine serum (HI-FBS;Gibco, A5669801), 1% glutaMAX (Gibco, 35050061) and 1% Penicillin/Streptomycin (Pen/Strep;Gibco, 15070063). A549 cells (ATCC, CCL-185) were maintained in DMEM containing 10% HI-FBS, 1% Pen/Strep, and 1% GlutaMAX. Immortalized equine lung epithelial cells (extEqFL, Applied Biological Materials (ABM), T0095) were propagated in PriGrow III medium (ABM, TM003) supplemented with 10% HI-FBS, 2mM L-glutamine (ABM G275) 600µg/mL G418 antibiotic (ABM, G271), and 1% Pen/Strep. Tissue culture flasks and plates for extEqFL cells were treated with applied extracellular matrix (ABM, G422).

## Hematology and serum biochemistry

Total white blood cell counts, white blood cell differentials, red blood cell counts, platelet counts, haematocrit values, total haemoglobin concentrations, mean cell volumes, mean corpuscular volumes, and mean corpuscular haemoglobin concentrations were analyzed from blood collected in tubes containing EDTA using a Vetscan HM5 laser based hematologic analyser (Zoetis). Serum samples were tested for concentrations of albumin, amylase, alanine aminotransferase (ALT), aspartate aminotransferase (AST), alkaline phosphatase (ALP), blood urea nitrogen (BUN), calcium, creatinine (CRE), C-reactive protein (CRP), gamma-glutamyl transferase (GGT), glucose, total protein, and uric acid by using a Piccolo point-of-care analyser and Biochemistry Panel Plus analyser discs (Abaxis).

## Histopathology and immunohistochemistry

Necropsies were performed on all subjects. Tissue samples from all major organs were collected for histopathologic and IHC examination, immersion-fixed in 10% neutral buffered formalin, and processed for histopathology. Relative severity scores for histological lesions and immunoreactivity were assigned by an American College of Veterinary Pathologists board-certified veterinary pathologist. Representative photomicrographs were qualitatively considered to display lesions that were nominally or ordinally measured by the masking of the veterinary pathologist after examination and ranking lesions to satiate the study objectives, as previously established.

Tissue sections were deparaffinized and rehydrated through xylene and graded ethanols. Slides went through heat antigen retrieval in a steamer at 95°C (203°F) for 20 minutes in Sigma Citrate Buffer, pH6.0, 10x (Sigma Aldrich, St. Louis, MO). The tissue sections were processed for IHC using the Thermo Autostainer 360 (ThermoFisher, Kalamazoo, MI). Specific anti-henipavirus N immunoreactivity was detected using an anti-Henipavirus N primary antibody at a 1:4000 dilution for 60 minutes. The secondary antibody used was biotinylated goat anti-rabbit IgG (Vector Laboratories, Burlingame, CA #BA-1000) at 1:200 for 30 minutes followed by Vector Streptavidin Alkaline Phosphatase at a dilution of 1:200 for 15 minutes (Vector Laboratories #SA-5100). Slides were developed with ImmPact Red Substrate Kit (Vector Laboratories #SK-5105) for 20 minutes and counterstained with haematoxylin for 30 seconds.

Tissue sections were deparaffinized and rehydrated through xylene and graded ethanols. Slides went through heat antigen retrieval in a steamer at 95°C (203˚F) for 20 minutes in Sigma Citrate Buffer, pH6.0, 10x (Sigma Aldrich, St. Louis, MO). To block endogenous peroxidase activity, slides were treated with a 3% hydrogen peroxide and rinsed in distilled water. The tissue sections were processed for IHC using the Thermo Autostainer 360 (ThermoFisher, Kalamazoo, MI). Sequential 15-minute incubations with avidin D and biotin solutions (Vector Laboratories, Burlingame, CA #SP-2001) were performed to block endogenous biotin reactivity. Specific anti-henipavirus N immunoreactivity was detected using an anti-henipavirus N primary antibody at a 1:4000 dilution for 60 minutes. The secondary antibody used was biotinylated goat anti-rabbit IgG (Vector Laboratories, Burlingame, CA #BA-1000) at 1:200 for 30 minutes followed by Vector Horseradish Peroxidase Streptavidin, R.T.U (Vector Laboratories #SA-5704) for 30 minutes. Slides were developed with Dako DAB chromogen (Dako, Carpenteria, CA #K3468) for 5 minutes and counterstained with haematoxylin for 30 seconds.

## Circulating chemokine/cytokine analysis

Circulating chemokines and cytokines from EDTA plasma specimens were measured utilizing LegendPlex bead-based immunoassays (BioLegend). Cross-species compatible (human, NHP) assay panels for analytes associated with inflammation, thrombosis, and chemokines/cytokines were performed following manufacturer instructions. Samples were analyzed in duplicate per-panel on a Cytek Aurora spectral flow cytometer. Raw data was processed using the LegendPlex Qognit data analysis software suite and concentration values were calculated based on standard curves. Subsequent analysis including log2 fold-change calculations and data visualization were performed in R v4.4.0 with ggplot2 v3.5.1.

## Virus titrations

Detection of replicating HeV-g2 and HeV-g1 in EDTA plasma and tissue specimens were performed by plaque assay on Vero cells. Briefly, samples were diluted in EMEM containing 2% HI-FBS and adsorbed in duplicate on confluent Vero cell monolayers in 6-well plates for 1 hour at 37°C (98.6˚F). Sample wells were overlayed with 0.8% agarose in 2X MEM and incubated at 37°C (98.6˚F), 5% CO_2_ for 48 hours. Plates were stained with 5% neutral red solution in PBS supplemented with 5% HI-FBS for 24h, after which stain was decanted, and plaques were quantified.

## Quantification of viral genomes

Determination of viral RNA copies from blood or tissue specimens was performed by quantitative reverse transcriptase-polymerase chain reaction (qRT-PCR) using primers/probe targeting the (F) gene of HeV-g1 (Redlands) or HeV-g2 (Gympie). Probe sequences for detection of HeV-g1 and HeV-g2 were 6FAM-5'CTCGGCTGTATCTGGAGCAGTCAC-3'-TAMRA (HeV-g1) and 6FAM-5’-TGCCCTGCCGGTAGTCTGACA-3’-TAMRA (HeV-g2) (Invitrogen). Primer sequences are available upon request. Cycle threshold (Ct) values representing copies of viral genomes were issued to calculate genome equivalents (GEq) of HeV-g1 or HeV-g2 based on a six-point standard curve. The standard curve consisted of serially diluted purified viral RNA extracted from the challenge virus stock of HeV-g1 or HeV-g2, and viral genomes were calculated using Avogadro’s number and the molecular weight of each viral genome. The LOD for each qRT-PCR assay to detect HeV-g1 or HeV-g2 was 1000 GEq per mL of fluid or per gram of tissue.

## Virus neutralization assays

The neutralizing titer of EDTA plasma specimens from HeV-g2-challenged subjects at sampled timepoints was determined by standard plaque reduction neutralization test (PRNT). Briefly, plasma samples were serially diluted and incubated with ~100PFU of HeV-g1, HeV-g2, or NiV-B for 1 hour at 37°C (98.6˚F). Sample dilution media (EMEM supplemented with 2% HI-FBS) was included alongside NHP samples to serve as an assay control. Individual samples were then plated in duplicate, and titers were determined as previously described. Percent neutralization was calculated by comparing the plaque counts of samples at each dilution to assay controls. Percent neutralization data was calculated in R v4.4.0. The 50% neutralizing antibody titer (NT50) was calculated in GraphPad Prism v10.4.1 via 4-parameter logistic regression of percent neutralization values.

## Viral replication kinetics

Briefly, Vero, A549, and extEqFL cells were seeded at 1.0E6 cells/per well in 6-well plates ~12h prior to infection with HeV-g2 or HeV-g1 which was diluted in normal growth medium for a final MOI of 0.01 per well. The inoculum was adsorbed to the cells for 1 hour at 37°C (98.6˚F), rocking every 15 minutes to distribute. Following adsorption, the inoculum was removed, and cell monolayers were washed 4x with PBS then 2mL of the basal medium for each cell line was added. Supernatants were collected at 1, 6, 12, 24, 36, 48, and 72 hours post-infection, aliquoted, and stored at -80˚C (-112˚F). All infections were performed in duplicate, and samples were titrated on Vero cells as previously described.

## ELISA

IgG antibody titers from serum samples of HeV-g2 challenged AGMs were determined using NHP species ELISA kits from Alpha Diagnostic International for anti-HeV-G IgG (ADI RV-501120-1) and anti-NiV-G IgG (ADI NiV-015) according to manufacturer instructions. Samples were assessed in duplicate on a BioTek Cytation 5 multimode plate reader. Blank wells were subtracted from all sample OD values to account for assay background. The average of the 1 U/mL Calibrator reagent supplied by the manufacturer was calculated, and all sample OD values greater than or equal to the 1 U/mL calibrator were recorded as positive. The endpoint titre is reported as the highest positive reciprocal serum dilution.

## Targeted transcriptomics

Whole blood samples containing HeV-g1 or HeV-g2 were inactivated in TRIzol LS (Invitrogen) or AVL buffer (Qiagen), and RNA was extracted following removal from BSL-4 containment. RNA isolation was performed according to manufacturer’s recommendation. Extracted RNA from TRIzol LS-inactivated samples was concentrated and DNAse digested with RNA Clean and Concentration-5 kit (Zymo Research). Purified RNA samples were hybridized with the NHP_Immunology_V2 reporter and capture codesets containing ~779 targets (Nanostring Technologies) for ~24h at 65˚C (149˚F) and subsequently loaded into nCounter microfluidics cartridge that was immediately run on a Nanostring nCounter SPRINT Profiler to determine fluorescent counts for each target.
